# Acute postcataract endophthalmitis at a referral center in northern Taiwan: Causative organisms, clinical features, and visual acuity outcomes after treatment: A retrospective cohort study

**DOI:** 10.1097/MD.0000000000008941

**Published:** 2017-12-08

**Authors:** Yi-Hao Chen, Jiann-Torng Chen, Ming-Cheng Tai, Yu-Ching Chou, Ching-Long Chen

**Affiliations:** a Department of Ophthalmology, Tri-Service General Hospital; b School of Public Health, National Defense Medical Center, Taipei, Taiwan.

**Keywords:** acute endophthalmitis, postcataract surgery

## Abstract

Acute-onset endophthalmitis after cataract surgery is a rare but serious complication. In this study, we identified the clinical profile of acute-onset endophthalmitis after cataract surgery using a retrospective review between January 1, 2009 and December 31, 2015 at a referral center in northern Taiwan. Twenty-five eyes of 25 patients with endophthalmitis were included; 60% were male. The mean age at diagnosis was 70.44 ± 1.66 years. The right eye was affected in 44% of patients. The mean time of cataract surgery to hospital was 12.76 ± 1.88 days. The mean time from the onset of endophthalmitis to hospital was 3.72 ± 0.78 days. Furthermore, 56% of patients received the immediate treatment of an intracameral or intravitreal injection of antibiotics at a clinic before referral. The mean initial visual acuity was 1.97 ± 0.15 logarithm of the minimum angle of resolution. The initial examination found corneal edema in 84% of patients, hypopyon in 48%, and vitritis in 88%. A total of 76% of patients underwent an initial treatment of pars plana vitrectomy and intraocular antibiotic injection (IOAI); 24% received an IOAI. The culture results of 52% (13/25) of patients were positive; 4 isolates were gram-positive, 8 were nontuberculosis *Mycobacterium abscessus*, and 1 was fungal. Thirteen of 25 patients had hypertension; 2 of 25 had diabetes mellitus. The mean follow-up time was 61.64 ± 8.27 days. Multiple factors influenced the final visual outcome of acute-onset endophthalmitis after cataract surgery. This study found that early referral for treatment could improve the final outcome. In addition, postcataract endophthalmitis caused by *M abscessus* recurs easily and has a worse visual outcome despite appropriate treatment.

## 1 Introduction

Acute-onset endophthalmitis is a rare but serious complication of cataract surgery that may lead to irreversible vision loss. The reported incidence rates range from 0.023% to 0.71%.^[1–3]^ In recent years, multiple preventive strategies for postcataract endophthalmitis have evolved. According to the results of the European Society of Cataract & Refractive Surgeons endophthalmitis study,^[4]^ prophylactic intracameral administration of antibiotics at the end of cataract surgery is common, and the rate of postcataract endophthalmitis has reduced. Although the risk of acute endophthalmitis is lower after antibiotic prophylaxis,^[4,5]^ the trend of the causative agents and associated outcomes may have changed due to the common use of prophylactic antibiotics and these changes can lead to functional loss of function of the eye.^[6]^

Although relatively rare, postcataract endophthalmitis presents a significant public health problem. Early diagnosis of acute endophthalmitis, immediate identification of the causative organism, and treatment with effective antibiotics are the keys to prevention of poor visual outcomes.^[7,8]^ According to the recommendations of endophthalmitis vitrectomy study,^[7]^ vitrectomy is most beneficial for patients with light perception (LP) vision, not for those with vision better than LP. The visual outcome of initial LP-only vision involving vitrectomy was a 3-fold chance of increase in visual acuity (VA) ≥ 20/40 and a 2-fold chance of increase in VA ≥ 20/100 compared to that on intraocular antibiotic injection (IOAI).^[7]^ However, several recent studies have reported the efficacy and advantages of early primary vitrectomy for postcataract endophthalmitis, and early primary vitrectomy has become one option for patients with VA better than LP.^[9,10]^

Given that the visual prognosis varies widely and depends largely on the etiologic organism,^[7,9,11]^ prompt diagnosis of the condition and early initiation of treatment are crucial to obtaining the best possible outcome. The purpose of this study was to analyze the current causative agents, clinical features, and visual outcomes associated with acute-onset postcataract endophthalmitis between 2009 and 2015 at a referral center. These findings may provide an empirical treatment strategy for endophthalmitis after cataract surgery.

## 2 Patients and methods

The study was approved by the Institutional Review Board of Tri-Service General Hospital (TSGHIRB no: 1-105-05-161). This was a retrospective chart review of patients with acute postcataract endophthalmitis between January 1, 2009 and December 31, 2015 at the Tri-Service General Hospital. The clinical records of patients who developed acute-onset postcataract endophthalmitis (within 6 weeks after cataract surgery), both culture-positive and culture-negative cases, were included in this study. Those who had undergone any combined surgery other than cataract extraction excluded. Patients with any underlying ocular disease other than cataract and delayed-onset endophthalmitis (6 weeks after cataract surgery) were excluded. Patients with traumatic or endogenous endophthalmitis were also excluded.

All patients with suspected acute endophthalmitis were clinically diagnosed; the disease was defined as marked intraocular inflammation within 6 weeks after cataract surgery, and/or proved by culture. Patients were examined and treated by a team of retinal specialists (Chief, Prof. Jiann-Torng Chen and Dr. Ching-Long Chen). When acute endophthalmitis was diagnosed, all patients received treatment based on the endophthalmitis vitrectomy study guidelines.^[7]^ Before any intraocular intervention, anterior chamber and vitreous specimens were collected by an aqueous and/or vitreous tap and/or vitrectomy, and specimens were cultured to identify the causative organism.

On the basis of the VA at the time of upon arrival at our hospital, patients immediately underwent either IOAI only or pars plana vitrectomy (PPV) and IOAI. Patients with LP vision or worse underwent PPV, whereas patients with hand motion (HM) vision or better underwent IOAI only. If a patient with VA better than LP met the following criteria, they still underwent both PPV and IOAI: diagnosis of diabetes mellitus or any systemic factors that may compromise immunological status, and presentation of toxic and fulminant signs, such as severe corneal edema, absence of light reflex, marked intraocular inflammation, and dense, nonclearing vitreous opacity. In addition, patients without these factors also underwent both PPV and IOAI if the surgeon thought it to be in the patient's best interest.

Initially, broad-spectrum antibiotics were administered intravitreally, topically, and systemically, and then adjusted on the basis of the culture results, antibiotic sensitivities, and clinical response. The antibiotics commonly used intravitreally included vancomycin, amikacin/ceftazidime, and amphotericin B, whereas the antibiotics administered systemically included gentamicin, cephazolin, and voriconazole. No systemic or intravitreal steroids were administered.

The following patient information was recorded: age, sex, involved eye, time of cataract surgery to our hospital, time from onset of signs and symptoms of endophthalmitis to our hospital, management before referral, initial VA upon arrival at our hospital, and intraocular pressure (IOP), baseline clinical characteristics (corneal edema, hypopyon, and vitritis), initial treatment upon arrival at our hospital, systemic disease (hypertension and/or diabetes mellitus), follow-up time, culture results, course of the disease (corneal abscess, retinal detachment, retinal hemorrhage, recurrence, removal of the intraocular lens (IOL), and repeated IOAI), and final VA. According to the endophthalmitis vitrectomy study,^[7]^ a final VA of ≥5/200 was chosen as the threshold of the visual outcome for data analysis. VA was recorded using the Snellen chart and converted to the logarithm of the minimum angle of resolution (logMAR) units. The nonnumerical VA was converted to logMAR notation as follows: counting fingers (CF), 2.0; HM, 2.3; LP, 2.7; no light perception (NLP), 3.0.^[12]^

### 2.1 Statistical analysis

We analyzed categorical variables as counts and percentages, which were compared using the Fisher exact test. Two continuous variables, age and time to presentation, were summarized using the mean and standard error and compared using a *t* test. Simple and multiple stepwise linear regression models were performed using the weight coefficient and the 95% confidence interval (CI) of the weight coefficient to evaluate factors that influenced final VA. All statistical analyses were performed using SPSS Statistics for Windows, Version 22.0 (IBM Corp., Armonk, NY); a *P*-value of .05 was considered significant.

## 3 Results

### 3.1 Clinical characteristics of patients

A total of 123 eyes of 123 patients with endophthalmitis were reviewed from January 2009 to December 2015. The medical records of 25 eyes of 25 patients were included in our study; data on 98 eyes of 98 patients were excluded. Of the 25 patients, 15 were male (60%). The mean age of the patients at diagnosis was 70.44 ± 1.66 years (range: 56–84 years). The right eye was affected in 11 of 25 patients (44%); in all others (56%), the left eye was affected. The mean time of cataract surgery to our hospital was 12.76 ± 1.88 days (range: 1–37 days).

All patients were referred from clinics in northern Taiwan. The mean time from the onset of endophthalmitis to our hospital was 3.72 ± 0.78 days (range: 0–14 days). A total of 14 patients (56%) had undergone immediate treatment with intracameral or intravitreal injection of antibiotics at their clinics before referral; others (44%) were referred without any intervention. Upon arrival at our hospital, the baseline visual acuity of 13 patients (52%) was ≥CF; 6 patients (24%) had a baseline of HM, and 6 patients (24%) had a baseline of ≤LP. The mean initial visual acuity was 1.97 ± 0.15 logMAR. The mean IOP upon arrival at our hospital was 15.21 ± 2.2 mm Hg (range: 4–48 mm Hg). In all, 18 of 25 patients (72%) had an IOP < 21 mm Hg, 5 (20%) had an IOP of ≥21 mm Hg, and the IOP of others (8%) was not recorded. At the time of upon arrival at our hospital, the initial examination found corneal edema in 21 of 25 patients (84%), hypopyon in 12 of 25 patients (48%), and vitritis in 22 of 25 patients (88%). A total of 19 of 25 patients (76%) underwent initial treatment with PPV and IOAI; 6 of 25 patients (24%) received IOAI. In systemic disease, 13 of 25 patients had hypertension and 2 of 25 had diabetes mellitus. The mean follow-up time was 61.64 ± 8.27 days (range: 5–123 days).

The clinical characteristics and demographic data of the patients compared with final VA are summarized in Table 1. Sex, time from onset of signs and symptoms of endophthalmitis to our hospital, management before referral, initial VA in logMAR, and follow-up time were significantly associated with final VA. Men had a significantly better final VA than women (*P* = .049). Patients with a shorter time from onset of signs and symptoms of endophthalmitis to our hospital had significantly better final VA (*P* = .001). Patients with management before referral had a significantly worse final VA than patients who were referred without intervention (*P* = .042). Patients with poor initial VA had significantly better final VA than those without (*P* = .045).

**Table 1:**
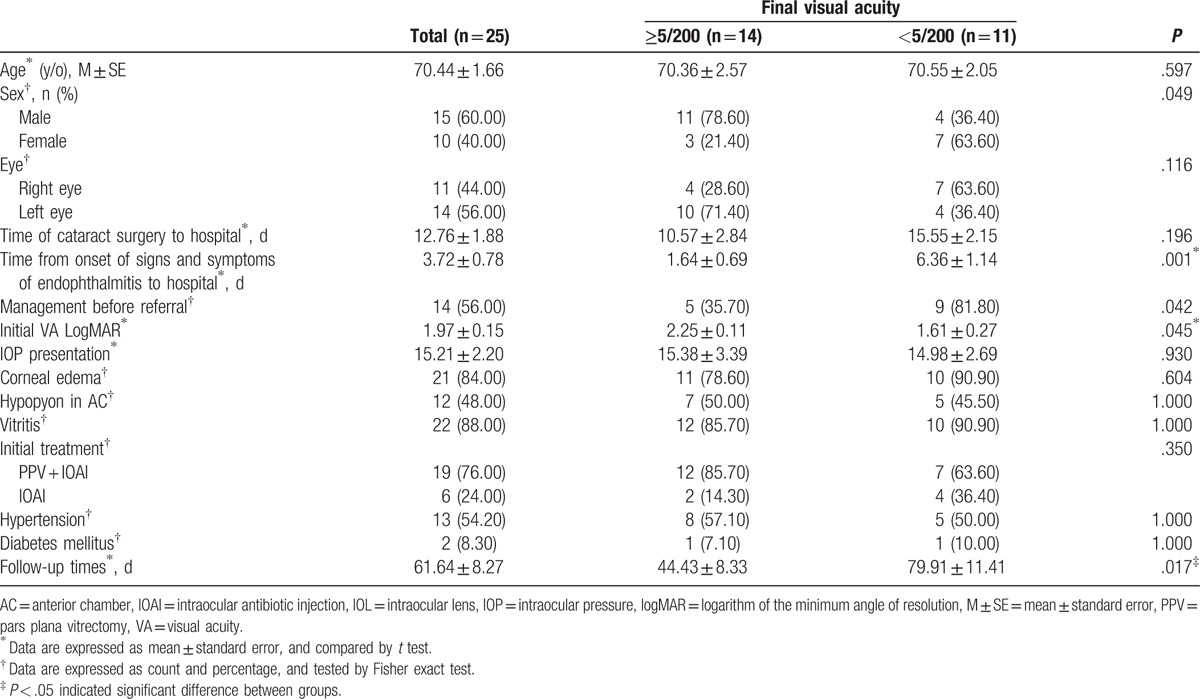
Summary of baseline characteristics by final visual acuity.

### 3.2 Microbiology evaluation

Among the 25 patients included in this study, 12 (48%) were culture-negative and 13 (52%) were culture-positive. Of the 13 positive cultures, 4 (30.8%) were gram-positive, 8 (61.5%) were nontuberculosis *Mycobacterium* (NTM), and 1 (7.7%) was fungal. The most common isolate was the nontuberculosis *Mycobacterium* spp., *Mycobacterium abscessus* (*M abscessus*, n = 8, 61.5%). The second most common were gram-positive bacteria (n = 4, 30.8%) including coagulase-negative *Staphylococcus* (n = 2, 15.4%) and *Enterococcus* species (n = 2, 15.4%). The last culture-positive case was a fungal infection, *Trichophyton* sp. (n = 1, 7.7%). All gram-positive bacteria were susceptible to vancomycin. *M abscessus* isolates were susceptible to amikacin. A comparison of the culture results with the final VA is shown in Table 2. Patients with *M abscessus* infections had worse final VA than those without (*P* < .001).

**Table 2:**
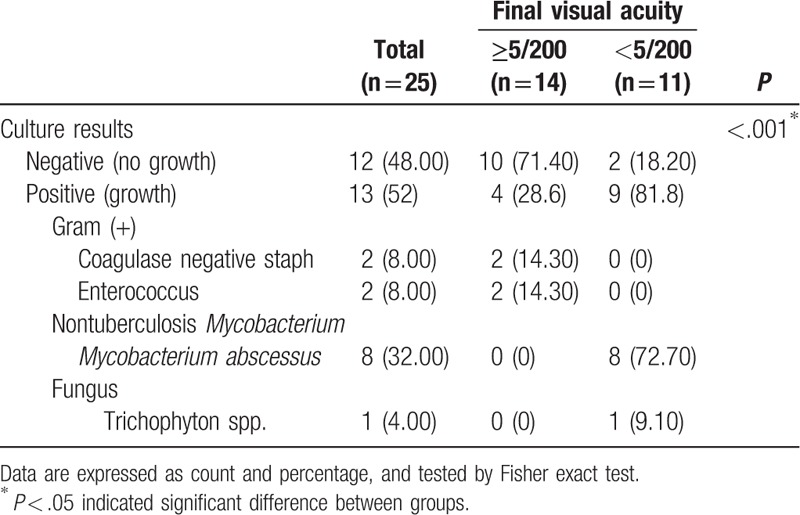
Summary of isolates by final visual acuity.

### 3.3 Course of the disease

During the follow-up after initial treatment, the clinical presentation showed 8 of 25 (32%) patients with a corneal abscess, 3 of 25 (12%) with retinal detachment, and 1 of 25 (4%) with retinal hemorrhage. The recurrence of signs of endophthalmitis was noted in 9 of 25 patients, who underwent PPV, removal of the IOL, and repeated IOAI. Among these 9 patients, 8 had *M abscessus* infections and 1 had a *Trichophyton* infection. The course of disease compared with final visual acuity is shown in Table 3. Patients with a corneal abscess, recurrent signs of endophthalmitis, removal of the IOL, and repeated IOAI had worse final VA than those without (*P* < .001).

**Table 3:**
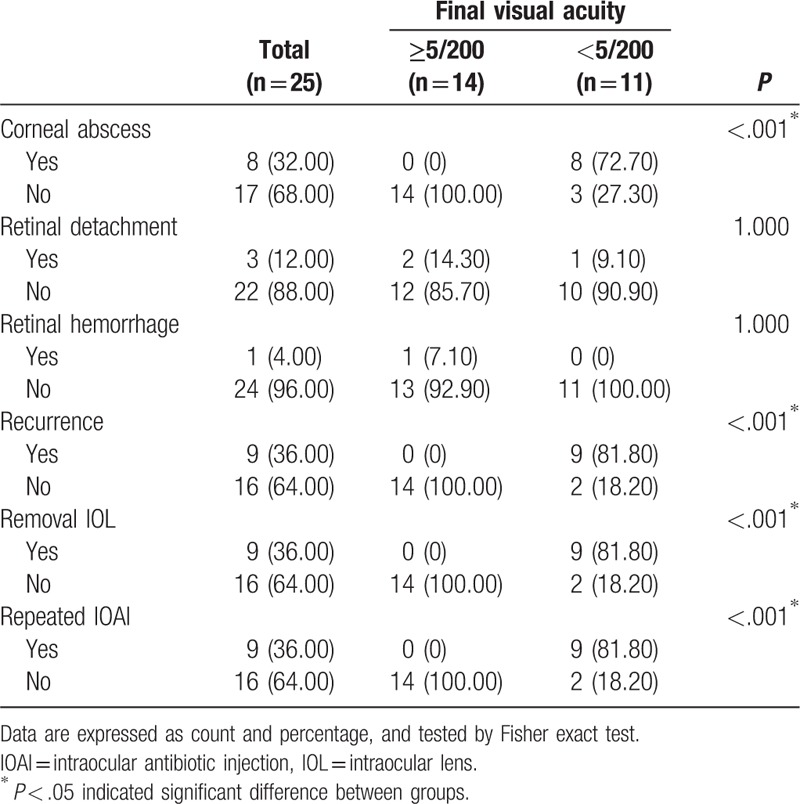
Summary the course of disease after initial treatment by final visual acuity.

### 3.4 Factors that influenced final VA

In the simple linear regression model, the baseline characteristics of time from onset of signs and symptoms of endophthalmitis to our hospital (days), management before referral, vitritis, and *M abscessus* infection were possible predictive factors of final VA. All variables were added stepwise to the multiple linear regression model, which revealed that 2 variables, vitritis and *M abscessus* infection, were significantly associated with poor final VA. Patients without vitritis had significantly better final VA than those with vitritis; the difference was −1.02 (95% CI: −1.78 to −0.26) logMAR. In addition, patients with *M abscessus* infection had significantly worse final VA than those without; the difference was 1.62 (95% CI: 1.16–2.08) logMAR. The predictive factors of final VA identified by simple and multiple regression models are shown in Table 4.

**Table 4:**
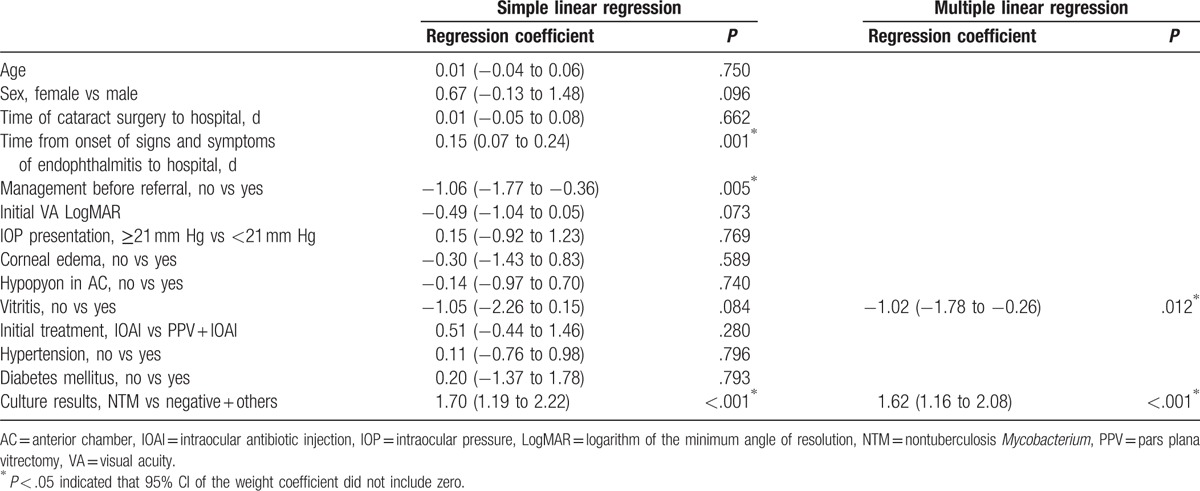
Simple and multiple linear regression models for final visual acuity (logMAR).

## 4 Discussion

Endophthalmitis after cataract surgery is a devastating complication, and many factors associated with final visual outcome have been reported. In this study, we found that sex is associated with visual outcome; men had better visual outcomes. The final visual outcome was also associated with a short time from onset of signs and symptoms of endophthalmitis to hospital. Cases that received treatment before referral had a significantly worse final VA than those referred without intervention. We also found that patients with poor initial VA had significantly better final VA. Herein, we report that patients with *M abscessus* infection had worse final VA than those without. Our findings also indicate that the disease course of patients with a corneal abscess, recurrent signs of endophthalmitis, necessary removal of the IOL, and repeated IOAI had a worse final VA than those without. In addition, a multiple linear regression model demonstrated that vitritis and *M abscessus* infection were independent factors that influenced final VA.

To date, there has been no consensus of the association between sex and final visual outcome after acute-onset postcataract endophthalmitis. Previous studies have reported that sex was not significantly related to final VA.^[13,14]^ Other studies have demonstrated that sex was significantly associated with final visual outcome.^[9,11]^ Consistent with second group of studies,^[9,11]^ our study showed a significant association between sex and final visual outcome.

Our study found an inverse relationship between the time from the onset of signs and symptoms of endophthalmitis to hospital and final VA, and this result suggests that early referral to hospital for further management resulted in better visual outcomes. Patients immediately received IOAI or PPV + IOAI, and the aqueous or vitreous humor was collected for culture. Appropriate treatment was selected according to the culture results and also improved the final visual outcome. Currently, if acute endophthalmitis is suspected, intracameral or intravitreal antibiotic injection is often performed at the clinic. This is why the number of cases of acute postcataract endophthalmitis has recently decreased, whereas the patients referred to hospital have usually received intracameral or intravitreal antibiotic injection at their clinic. As these patients had endophthalmitis that was more difficult to control, previous treatment at the clinic could interfere with the culture results and delay appropriate treatment after broad-spectrum antibiotic treatment. This could explain why management before referral had significantly worse final VA than referral without intervention.

In addition, previous studies have shown that the final visual outcome was associated with the initial VA.^[7,8,15]^ The endophthalmitis vitrectomy study reported that only the initial visual acuity of LP is an independent risk factor for a decreased final VA.^[7]^ A French group^[8]^ and an United Kingdom group^[15]^ have reported that poor VA at presentation is a significant factor associated with poor visual outcome. In the present study, we found that the initial VA was 2.25 ± 0.11 logMAR at final VA ≥ 5/200 group and 1.61 ± 0.27 at final VA < 5/200 group, and there was an inverse relationship between the initial and finial VA. This result differs from that of previous studies.^[7,8,15]^ One possible explanation is the difference in sample size. In our series, only 5 of 25 (20%) patients presented with a VA of 5/200 or better; all others (80%) had a VA < 5/200 upon arrival at our hospital. Another explanation is that all patients with a VA < 5/200 upon arrival at our hospital in this study underwent PPV + IOAI. Immediate PPV might directly remove the causative organism and inflammatory material, as well as improve the final visual outcome.

Endophthalmitis is a rare, sight-threatening complication of cataract surgery, and the most serious complication of the procedure. Organism isolation is the basis for treatment. Previous studies reported that gram-positive bacteria, especially coagulase-negative *Staphylococcus*, were the most frequently isolated bacteria.^[1,7,16]^ In our study, only 4 of 13 culture-positive results were gram-positive bacteria, including 2 coagulase-negative *Staphylococcus* and 2 *Enterococcus*. In contrast, 8 of 13 culture-positive results were a nontuberculous *Mycobacterium*, *M abscessus*. These findings could have 2 possible causes. First, in 2007, the European Society of Cataract & Refractive Surgeons endophthalmitis study reported the effect of prophylactic antibiotics in reducing the rate of endophthalmitis after cataract surgery.^[4]^ Prophylactic antibiotics have been commonly used in our country, and the number of postcataract endophthalmitis cases decreased between 2009 and 2015 compared to reports between 2002 and 2008.^[11]^ Second, one outbreak of postcataract endophthalmitis caused by *M abscessus* came from the same clinic in northern Taiwan, and our group treated 8 patients with *M abscessus* endophthalmitis from this clinic.

The final visual outcome has also been reported to be associated with the causative organism.^[7,8]^ Previous studies have shown that gram-positive infections^[7,8]^ and culture-negative cases^[8,17]^ have better final visual outcomes after treatment, whereas NTM infections have worse final visual outcomes.^[18]^ In the present study, the final visual outcome showed that 71.4% of culture-negative cases and all gram-positive cases had good visual outcomes (VA ≥ 5/200), whereas all NTM infections had poor visual outcomes (VA < 5/200). These results are similar to those of previous reports.^[7,8,17,18]^ In addition, a previous study reported that NTM infection trends toward a poor final visual outcome, despite appropriate treatment based on culture results.^[19]^ Similar to a previous study,^[19]^ our series found that patients with *M abscessus* infection experienced recurrent signs of endophthalmitis and improved after repeated IOAI and removal of the IOL, whereas the final VA was very poor.

In the present study, patients with corneal abscess, recurrent signs of endophthalmitis, necessary removal of the IOL, and repeated IOAI had poor visual outcomes. These patients had endophthalmitis due to a fungal or NTM infection. Patients with NTM endophthalmitis experienced a corneal abscess, a sight-threatening complication of endophthalmitis that could induce corneal scars or perforation of the eyeball.^[20]^ Additionally, previous studies have reported that endophthalmitis caused by fungal or NTM infections could form biofilms, which increased the resistance to conventional antibiotics, the need for removal of the IOL, and repeated IOAI.^[20,21]^ Therefore, similar to that in previous studies,^[20,21]^ the patients with endophthalmitis caused by fungal or NTM infection in our study underwent removal of the IOL and repeated IOAI after appropriate treatment and had poor visual outcomes.

The simple linear regression of our series identified that the time from onset of signs and symptoms of endophthalmitis to hospital, management before referral, and NTM infection were significantly related to the final visual outcome. A multiple linear regression found that vitritis and NTM infection were independent factors that affected the final visual outcome. Previous studies have reported that patients with a cloudy vitreous at presentation had worse visual outcomes^[7,8]^ and that patients with NTM infection usually had vitritis and worse visual outcomes.^[22–25]^ The results of our series are similar to those in previous reports.^[7,8,22–25]^ Moreover, NTM are ubiquitous in the environment in soil, dust, and water, and can cause infections of the external adnexal and ocular tissues.^[20]^*M abscessus* belongs to the rapidly growing NTM group and has been implicated in the etiology of endophthalmitis after cataract surgery.^[20]^ Endophthalmitis caused by *M abscessus* is rare but is generally associated with poor VA outcomes.^[22–25]^ In our study, only one patient had 2/200 VA after the final follow-up; all others experienced loss of vision after treatment. Consistent with previous reports,^[22–25]^ our series showed that the visual outcomes of *M abscessus* infection were generally poor even after treatment.

The limitations of this study include the retrospective chart review design and the inclusion of a single center, as the study may lack data on variables such as medical records before surgery, times of cataract surgery, intraoperative complications, and treatment before referral by the clinic. Thus, the present study could not assess the relationship between these variables and the final VA. Furthermore, the sample size of the present study was small, which may introduce bias and limit the statistical significance. Therefore, further study with a larger number of patients is needed to validate our findings.

In conclusion, our study demonstrated that sex, a shorter time from onset of signs and symptoms of endophthalmitis to hospital, and poor initial VA are associated with a good visual outcome. Vitritis and *M abscessus* infection are independent factors of final VA. Postcataract endophthalmitis caused by *M abscessus* recurs easily and is associated with worse visual outcomes despite appropriate treatment.
